# Spatiotemporal distribution of *Mycobacterium ulcerans* and other mycolactone producing mycobacteria in southeastern United States

**DOI:** 10.1080/22221751.2025.2521853

**Published:** 2025-06-17

**Authors:** Magdalene Dogbe, Cody Roberts, Kayla M. Fast, Alex W. Rakestraw, Joseph P. Receveur, Katherine Yoskowitz, Jennifer L. Pechal, Michael W. Sandel, Christine Chevillon, Jean-François Guégan, Mark E. Benbow, Heather R. Jordan

**Affiliations:** a Department of Biological Sciences, Mississippi State University, Starkville, MS, USA; b Department of Wildlife, Fisheries and Aquaculture, Mississippi State University, Starkville, MS, USA; c Department of Biological and Environmental Sciences, The University of West Alabama, Livingston, AL, USA; d Department of Entomology, Michigan State University, East Lansing, MI, USA; e Forest and Wildlife Research Center, Mississippi State University, Mississippi, MS, USA; f MIVEGEC, UMR CNRS, IRD, Université de Montpellier, Montpellier, France; g Epidémiologie des maladies animales (UMR EPIA), INRAE, VetAgro Sup, Saint-Genès-Champanelle, France; h Ecology, Evolution and Behavior Program, Michigan State University, East Lansing, MI, USA; i AgBioResearch, Michigan State University, East Lansing, MI, USA; j Department of Osteopathic Medical Specialties, Michigan State University, East Lansing, MI, USA

**Keywords:** *Mycobacterium ulcerans*, *Mycobacterium liflandii*, mycolactone-producing mycobacteria, Buruli Ulcer, Louisiana, Alabama, Mississippi, Southeastern United States

## Abstract

Buruli ulcer (BU) is a chronic and debilitating skin disease caused by the environmental pathogen, *Mycobacterium ulcerans* (MU). The primary virulence determinant is mycolactone, a cytotoxic lipid compound unique to MU and its other mycolactone producing mycobacteria (MPM) ecological variants. Although BU prevalence is highest in West Africa and Australia, little is known about MU and other MPM distribution in non-endemic regions such as the Southeastern United States (US). In this study, environmental samples (water filtrand, plant biofilm, soil, aquatic invertebrates) were collected from nine freshwater sites across Louisiana, Mississippi and Alabama over three sampling periods (August 2020, November 2020, March 2021). Samples were screened for MU and MPM presence and abundance by PCR and genotyped using variable number tandem repeat (VNTR) profiling. All nine sites were positive for MU or other MPM DNA in at least one substrate, except invertebrates. Overall, mean concentrations were 4.3 × 10^4^ genome units (GU)/sample in August 2020, 1.26 GU/sample in November 2020, and 55.5 GU/sample in March 2021. Profiling by VNTR identified four MU (designated A-D) and one *M. liflandii* genotype(s), among environmental samples, with genotype frequencies varying by site and sampling time. Detection of MU and *M. liflandii* genotypes in Southeastern US aquatic environments, matching those from BU endemic regions, provides rationale for ongoing surveillance. Our findings broaden the known geographic range of MU and MPMs and offer baseline data to help predict and prevent and predict the possibility of zoonotic transmission in Southeastern US.

## Introduction

*Mycobacterium ulcerans* (MU) is an environmental pathogen that causes a debilitating human skin disease known as Buruli ulcer (BU) [1]. *M. ulcerans* possesses the ability to cause tissue damage and dampening of the immune response due to production of a cytotoxic, lipid compound called mycolactone [1-3]. Over the years, additional mycobacteria have been identified synthesizing unique mycolactone congeners. These mycolactone producing mycobacteria (MPM), including MU, belong to a sub-group of non-tuberculosis mycobacteria (NTMs) that cause disease in a range of hosts including humans and other vertebrates [4]. MPMs are closely related phylogenetically, though differ in known host ranges [4]. Variable number tandem repeat (VNTR) profiling of MPMs has also revealed genetic and geographic diversity of isolates and environmental samples [5-7]. For example, VNTR genotyping of MU from patients in West Africa revealed multiple, distinct clonal complexes (designated A, B, C, D, etc.) in different communities in Ghana and Côte d’Ivoire [3].

BU is reported in at least thirty-three countries, with prevalence highest in West Africa and Australia [1]. The most described risk factor for BU and other MPM infections is exposure to natural, contaminated aquatic habitats [8]. In endemic regions of West Africa and Australia, MU has been detected in fish, aquatic and terrestrial plant biofilms, water filtrand, soil, mosquitoes and other insects [5,9–11]. Despite these findings, potential MU reservoirs and transmission mechanisms are still under investigation and may vary by geographical location and environmental conditions. Notably, no BU cases have been reported originating in the US [12]. However, BU has been documented in neighbouring areas such as Mexico and other Neotropical countries [13]. A prior environmental study in 2013 detected presumptive MU and MPM DNA in aquatic samples from southern Louisiana [14], suggesting the bacterium’s presence in US environments even in the absence of human cases. They also identified seasonal variations in detection, with a decrease in positive samples in fall compared to winter each year. However, their study was limited to Southern Louisiana and a limited number of MU molecular targets. Those targets, including IS*2404*, MUL_0999, MUL_2832, MUL_3218, are either not specific for MPMs or not present in all MPMs, and not all targets were identified within the same samples, decreasing the strength of the results [6,12]. Our present study is unique as it reports MU/MPM detection in three US states: Alabama, Mississippi and Louisiana where we used qPCR targeting the enoyl reductase (ER) domain from polyketide synthase genes encoding mycolactone to absolutely quantify MPM genomic units and VNTR profiling for MU/MPM strain differentiation [4]. *M. ulcerans*/MPM VNTR loci exhibit a high degree of polymorphism due to variations in the number of repeat units. This variation between bacterial strains allows for fine-scale differentiation, even among closely related isolates. Further, analysing multiple VNTR loci significantly increases the discriminatory power, creating unique “fingerprints” for individual strains and is a means to discriminate between mycobacteria (and other bacterial) species and strains. Thus, we can, with a higher degree of certainty, identify MU/MPM in the Southern US across our three sampled states. Detecting MU/MPMs in environmental samples within the US has significant implications for understanding the bacterium's ecology, potential public health risks (even if currently low), and the health of aquatic wildlife. Furthermore, this finding opens new avenues for research into this neglected tropical disease in a non-endemic setting [9,15-24].

## Materials and methods

### Site selection

**Figure 1.**
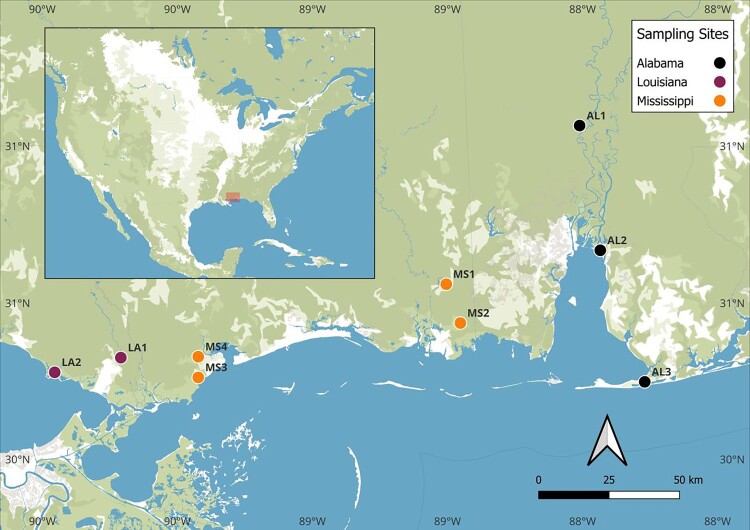
Map of sites sampled in August 2020, November 2020 and March 2021.

Nine sampling sites were selected in Louisiana (LA), Mississippi (MS), and Alabama (AL) based on similarities to known MU positive sites described in other parts of the world [5,9] (Figure 1 and Figure S1). These criteria included slow-flowing waterbodies, often isolated riverine wetlands or along backwater habitats of slow-moving rivers and streams and draining wetlands. Such conditions have been associated with MU presence. Specifically, the Louisiana sites (LA1 and LA2) were chosen based on locations where MU had been previously detected by Hennigan et al. [14], ensuring continuity with that study. Other sites (MS1 – MS4 and AL1 – AL3) were selected via QGIS web map survey of wetlands, targeting areas with swampy characteristics or slow-flowing bayous. Alabama site 1 (AL1) is a small stream feeding into the Mobile River and samples were collected near a boat landing. Alabama site 2 (AL2) is a wetland of the Mobile Bay estuary located around the Meaher State Park and Alabama site 3 (AL3) is a site under a bridge located near Fort Morgan Road trail in Alabama. Mississippi Site 1 (MS1) is a small pond found along Hardy Waltman road in Moss Point, Mississippi. Mississippi site 2 (MS2) is a riverbank of the Escatawpa River, Mississippi site 3 (MS3) is a site under a bridge and adjacent to a railway, and Mississippi site 4 (MS4) is a wetland connecting to the St. Louis Bay. Figure 1 represents the geographical locations, with images of sites shown in Supplemental Figure S1 and coordinates listed in Table 1. Permission for private land access was obtained when applicable. All nine sites were sampled in August 2020 and March 2021; however, due to limitations with access, only six sites (excluding MS1, MS4, AL3) were sampled in November 2020.

**Table 1.** GPS Coordinates of sampling sites.

State	Site ID	Site Name	Latitude	Longitude
Alabama	AL1	Cedar Creek Landing	31.065116	−88.011116
Alabama	AL2	Meaher State Park	30.6684453	−87.934463
Alabama	AL3	Fort Morgan Rd	30.248285	−87.770062
Mississippi	MS1	Hardy Waltman Road	30.560335	−88.504713
Mississippi	MS2	Escatawpa River	30.436349	−88.452459
Mississippi	MS3	Railroad Ave	30.262561	−89.422771
Mississippi	MS4	Riverview Dr	30.32891	−89.422771
Louisiana	LA1	Davis Landing Road	30.3258264	−89.709333
Louisiana	LA2	Lacombe	30.278842	−89.9543

### Environmental Sample Collection

Macrophyte biofilms, soils, water column suspended solids, and aquatic macroinvertebrates were sampled at each site. Macrophyte biofilms were sampled in triplicate from the three most dominant aquatic plant species, including roots, stems, and leaves using our established methods for MPM detection [9]. Plant clippings were placed in sealable, sterile plastic bags and 50 mL of sterile water added. Macrophyte biofilms were dislodged by vigorously rubbing samples within the bag for 1 min and then 25 mL of liquid suspension was placed into a 50 mL screw cap tube and preserved with 25 mL RNALater™. Soil samples were collected in triplicate from edges of the waterbodies (5.0 grams/triplicate) and preserved in 15 mL screw-cap tubes containing 5 mL of RNALater™. Six or seven water filters were collected by first passaging 50 mL of water through a 25 mm fibre glass (1.6μm) then through a 25 mm nitrocellulose (0.2μm) filter. Filters were preserved with a few drops of RNALater™ and placed into sealable plastic bags. In August 2020, representative aquatic macroinvertebrates were sampled using our established standardized techniques that have been used in previous MPM assays [9,25]. Macroinvertebrates were identified to family using Merritt, Cummins and Berg [26] at Michigan State University. We did an external surface decontamination to remove nucleic acids and cells from the surface of the macroinvertebrates prior to DNA extraction.

### DNA Extraction and PCR for detection of the presence and abundance of MPMs

DNA extraction was performed using reagents and methods previously described and detailed as supplementary material [6]. To verify DNA extraction efficiency and account for inhibitors, one filter from each filter set was spiked with MU bacilli as a positive matrix control and was included with remaining filter sets for DNA extraction and downstream analyses. DNA was subjected to qPCR using a Taqman probe with VIC fluorophore and primers and standards targeting ER as previously described and detailed as supplementary material [9]. Samples where MPMs were detected were further subjected to VNTR profiling targeting four loci: MIRU1, locus 6, ST1, and locus 19 using previously described methods and detailed as supplementary material [9]. Strain and regional specific controls were used for comparison and bands were confirmed with Sanger Sequencing from Arizona State University.

### Statistical Analyses and MST Analysis

Statistical analyses were performed using GraphPad Prism version 7 and IBM SPSS Statistics for Macintosh, version 29.0.1. The Shapiro-Wilk’s test determined non-normal distributions across all comparisons. Kruskal–Wallis with Bonferroni correction was used to determine significant differences of positivity and concentration across matrix, location and sampling time. Concentration analyses excluded zeros where statistical differences in concentration magnitude were analysed from positive data. Logistic regression was used to determine if there was a statistically significant interaction effect between location and sampling time associated with positivity. Concentration data was modelled using a generalized linear model (GLM) of square root transformed data with a gamma distribution and log link function. Location, sampling time, and their interaction were included as predictors for concentration magnitude among positive samples. Significance was determined by *p*-value (or adjusted *p*-value) ≤ 0.005. Pearson correlation between MPM positivity and abiotic factors (temperature, pH, conductance) were also examined. A Minimum spanning tree (MST) was built based on VNTR profiles of samples and geographically distinct isolates, as well as published profiles [5,16] using RStudio (RStudio Team, 2024, v2024.12.1-563). The R package *igraph* version 2.1.4 was used to perform the MST analysis and the final visualization was drawn with *ggraph* version 2.2.1.

## Results

### Seasonal Distribution of MPMs Across Sites

**Figure 2.**
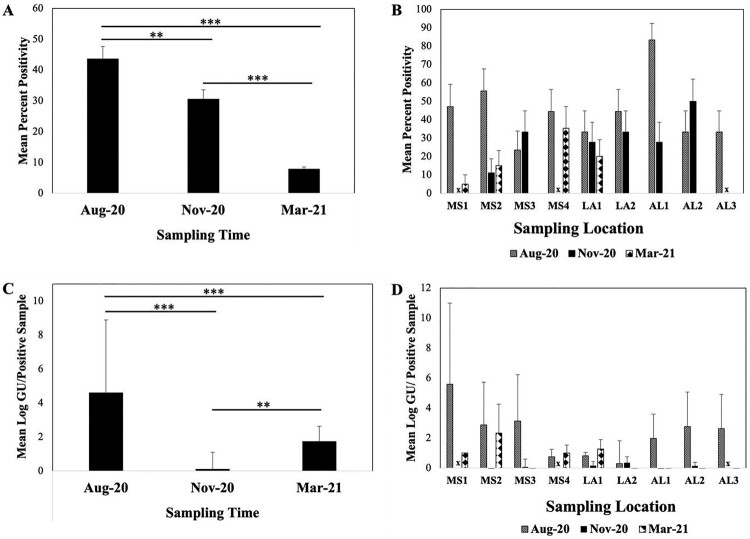
Mycolactone producing mycobacteria mean positivity (A) and concentration (C) across sampling time. Sample positivity according to site and time and mean concentration (mean log10 GU of positive samples are shown in panels B and D. MS1: Mississippi Site 1; MS2: Mississippi Site 2; MS3: Mississippi Site 3; LA1: Louisiana Site 1; LA2: Louisiana Site 2; AL1: Alabama Site 1; AL2: Alabama Site 2; AL3: Alabama Site 3. x = Sites not sampled due to limited accessibility. Error bars denote standard error of the mean. *** represent *p* values <0.001, and ** represent *p* values <0.01.

A total of 447 samples (excluding invertebrates) were collected and analyzed for MPM presence and abundance (Table 2). Of these, 119 (26.6%) were ER positive indicating presumptive MPMs. There was a significant difference associated with positivity and sampling time (F_2,447_ = 57.300; *p* < 0.001). Samples assayed in August 2020 had significantly higher positivity compared to November 2020, and March 2021 (p_Bonferroni adj. _= 0.046, and p_Bonferroni adj_ < 0.001, respectively) and there was significant decrease in MPM % positivity from November 2020 to March 2021 (Figure 2A, p_Bonferroni adj_ <0.001). All nine sampled sites were positive for MPMs for one or more matrices in August 2020 where highest positivity was recorded from AL1 (83.3%; ± SD = 38.3), followed by MS2 (55.5%; ± SD = 51.1); MS4 and LA2 (both 44.4%; ± SD = 51.1); MS1 (47.1%; ± SD = 51.4); AL2, AL3, and LA1 (all 33.3%; ± SD = 48.5); and MS3 (23.5%; ± SD = 43.7) (Figure 2B). There were significant differences identified in concentrations and sampling time (F_2,447_ = 63.789; *p* < 0.001). Highest MPM concentrations were detected in August 2020 (4.60 mean log GU/sample, ± SD = 5.40), followed by March 2021 (1.74 mean log GU/sample, ± SD = 2.0), then November 2020 (0.10 mean log GU/ sample, ± SD = 0.01). Significant differences in concentration were found between August and November 2020 (p_Bonferroni adj_ <0.001), August 2020 and March 2021 (p_Bonferroni adj_ <0.001) and November 2020 and March 2021 (p_Bonferroni adj_ = 0.006).

**Table 2.** MPM per cent positivity and mean concentration (mean genome units/positive sample) across sample matrices and time. Values into brackets mean percentage of positivity.

	August 2020	November 2020	March 2021	All Timepoints
*Water filters*				
Positivity (%)	67/108 (62.0)	31/70 (44.3)	14/126 (11.1)	114/306 (37.3)
Mean MPM concentration	364.2	1.26	55.5	221.2
*Soil*				
Positivity (%)	3/27 (11.1)	0/18 (0)	0/24 (0)	3/69 (4.35)
Mean MPM concentration	1.3 × 106	0	0	1.3 × 106
*Macrophyte biofilm*				
Positivity (%)	2/27 (7.41)	0/18 (0)	0/27 (0)	2/72 (2.8)
Mean MPM concentration	22.6	N/A	N/A	22.6
*Macroinvertebrates*				
Positivity (%)	0/188 (0)			
Mean MPM concentration	N/A			

In August 2020, highest concentrations were detected among positive samples from MS1 (5.86 mean log GU/sample; ± SD = 5.59), followed by MS2 (2.87 mean log GU/sample; ± SD = 3.34), MS3 (3.14 mean log GU/sample; ± SD = 3.44), AL2 (2.76 mean log GU/sample; ± SD = 2.68), and AL3 (2.63 mean log GU/sample; ± SD = 2.67) (Figure 2D). Sites AL1, LA2, MS4, and LA1 had considerably lower MPM concentrations with 1.97 (± SD = 2.21), 0.30 (± SD = 1.98), 0.75 (± SD = 0.95), and 0.81 (± SD = 0.62) mean log GU/sample, respectively overall (Figure 2D).

Only six of the nine sites were sampled in November 2020 due to limited accessibility, but all six were positive with AL2 measuring highest positivity (50.0%; ± SD = 51.4) followed by MS3 and LA2 (both 33.3%; ± SD = 48.5), LA1 and AL1 (both 27.8%; ± SD = 46.1), and MS2 (11.1%; ± SD = 32.3). The six sites sampled in November 2020 showed decreased concentrations compared to August 2020 sampling (Figure 2B) where mean log GU/sample across sites from all positive samples were 0.35 mean log GU/positive sample or less.

Only four of nine sites sampled in March 2021 had ER positive samples with highest positivity recorded from MS4 (35.3%; ± SD = 49.3), followed by LA1 (20.0%; ± SD = 41.0), MS2 (15.0%; ± SD = 36.6), and MS1 (5.0%; ± SD = 22.4) (Figure 2B). All samples assayed from MS3, LA2, and AL1-3 were negative. The highest mean concentrations from positive samples were from MS2 (2.32 mean log GU/sample; ± SD = 2.18), followed by LA1 (1.27 mean log GU/sample; ± SD = 0.93), MS4 (1.00 mean log GU/sample; SD = 0.81) and MS1 (1.00 mean GU/sample; ± SD = 0.92).

The overall binary logistic regression model was significant (χ² (2) = 60.526, *p* < 0.001) but Hosmer-Lemeshow suggested a poor model fit (χ² (8) = 27.834, *p* < 0.001). Nagelkerke's R² indicated that the model explained 18.5% of the variance in the likelihood of a positive sample. Sampling time significantly predicted the likelihood of a positive sample (Wald χ2(df = 1) = 5.158, *p* = 0.023) where the odds ratio for the interaction term was 0.501 (95% CI [0.276, 0.910]). Neither location nor the interaction between location and sampling time significantly predicted the likelihood of a positive sample. However, the generalized linear model showed that location (Wald χ^2^ (df  = 8) = 40.978, *p* < 0.001), sampling time (Wald χ^2^ (df  = 2) = 157.552, *p* < 0.001) and the interaction between location and sampling time (Wald χ^2^ (df  = 8) = 57.116, *p* < 0.001) were significant predictors of concentration magnitude among positive samples.

### Detection of environmental MU/MPMs was highest among assayed water filters

**Figure 3.**
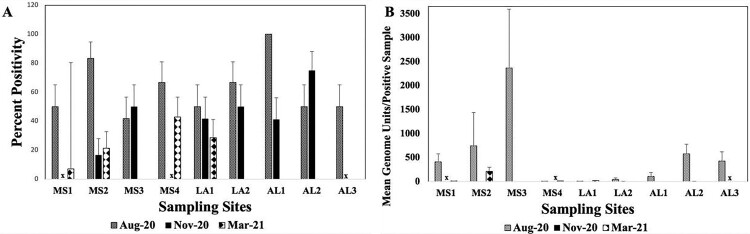
Mean Positivity (A) and Concentration (B) of positive water filter samples per Site and sampling time. Concentration was expressed as mean genome units across positive samples per site and sampling time. x = Sites not sampled due to limited accessibility. Error bars are standard error of the mean.

Four different matrices (water filters of suspended solids, soil, macrophyte biofilm, macroinvertebrates) were assayed to determine the presence and abundance of MPMs in sampled sites over time. There was no difference in positivity or concentration between filter types so data from both filter types were combined. There was a significant difference in positivity according to matrices (F_2,447_ = 58.72; *p* < 0.001). Data showed MPM positivity driven by water filter samples (Table 2) with significantly higher % positivity than soil or macrophyte biofilms (p_Bonferroni adj_. < 0.001 for both); and there were no positives for aquatic macroinvertebrates. Water filters were positive across all sites and sampling times, with significantly higher positivity across sampling times (F_2,298_ = 64.93; *p* < 0.001, Figure 3A). Bonferroni post hoc testing showed positivity trending towards significant in August 2020 (62.03%, ± SD = 48.8) compared to November 2020 (44.30%, SD = 50.0; *p* = 0.058) and March 2021 (11.1%, ± SD = 31.5; *p* < 0.001). Per cent positivity decrease from November 2020 to March 2021 was also significant (*p* < 0.001, Table 2, Figure 3A). In August 2020, highest MPM positivity was detected from AL1 water filters (100.0%, ± SD = 0.0), followed by MS2 (83.3%, ± SD = 38.9), MS4 and LA2 (both 66.7%, ± SD = 49.2), LA1, MS1, AL2 and AL3 (all 50.0%, ± SD = 52.2), and MS3 (41.7%, ± SD = 51.5, Figure 3A). Overall concentrations were low, with highest MPM concentration detected from MS3 (1.4 x10^3^ mean GU/sample, ± SD = 2.7 × 10^3^), followed by MS2 (743.1 mean GU/sample ± SD = 2.2 × 10^3^), AL2 (577.2 mean GU/sample, ± SD = 485.7), AL3 (428.2 mean GU/sample, ± SD = 467.9), and MS1 (408.8 mean GU/sample, ± SD = 405.9). Sites LA1 and LA2, and MS4 all had positive samples less than 50.0 mean GU/sample (± SD = 4.17, 95.8, and 7.66, respectively, Figure 3B).

Of sampled sites in November 2020, AL2 had the highest positivity from water filters (75.0%, ± SD = 45.2), followed by LA2 and MS3 (both 50.0%, ± SD = 52.2), LA1 and AL1 (both 41.7%, ± SD = 51.5), and MS2 (16.7%, ± SD = 38.9), though all positive samples had concentrations less than 2.5 mean GU/sample. Only 4 of the 9 sites had positive water filters in March 2021: MS4 (42.9%, ± SD = 51.4), LA1 (28.6%, ± SD = 46.9), MS2 (21.9%, ± SD = 42.6), and MS1 (7.1% ± SD = 2.74) (Figure 3A). Concentrations of positive samples were low for all four sites with highest from MS2 (210.7 mean GU/sample, ± SD = 129.3). MS1, MS4, and LA1 all had positive concentrations that were less than 20.0 mean GU/sample (Figure 3B). Overall, there were no significant differences among overall locations in either positivity or concentration, but filter positivity differences were significant across sampling time (F_2,306_ = 67.263; *p* < 0.001), with significant differences between August 2020 and March 2021 (p_Bonferroni adj._<0.001), and between November 2020 and March 2021 (p_Bonferroni adj._<0.001). Filter concentrations differences were also significant across sampling time (F_2,306_ = 71.714; *p* < 0.001), with significant differences between August 2020 and November 2020 (p_Bonferroni_ adj. < 0.001), August 2020 and March 2021 (p_Bonferroni adj._<0.001), and between November 2020 and March 2021(p_Bonferroni adj. _= 0.005).

The overall binary logistic regression model was significant (χ²(3) = 70.576, *p* < 0.001) but Hosmer-Lemeshow suggested a poor model fit (χ²(8) = 27.784, *p* < 0.001). Nagelkerke's R² indicated that the model explained 28.1% of the variance in the likelihood of a positive sample. Sampling time significantly predicted the likelihood of a positive sample (Wald χ2(df = 1) = 5.978, *p* = 0.014) where the odds ratio for the interaction term was 0.435 (95% CI [0.223, 0.848]). Neither location nor the interaction between location and sampling time significantly predicted the likelihood of a positive sample. However, the generalized linear model showed that location (Wald χ^2^ (df  = 8) = 51.567, *p* < 0.001), sampling time (Wald χ^2^ (df  = 2) = 191.014, *p* < 0.001), and the interaction between location and sampling time (Wald χ^2^ (df  = 8) = 48.678, *p* < 0.001) were significant predictors of filter concentration magnitude among positive samples.

Only three soil samples from the August 2020 sampling were positive over the entire sample period (Table 2). Two of the three were from MS1 with concentrations of 1.54 × 10^6^ and 1.57 × 10^6^ GU/sample, respectively. The third soil sample was from AL1 with a concentration of 211.0 GU/sample. Only two macrophyte biofilm samples were positive overall and both were from AL1 sampled August 2020 with concentrations of 11.2 and 33.98 GU/sample, respectively.

Of 188 specimens representing 59 taxonomic families sampled, there were no MPM detections among aquatic macroinvertebrates collected at any site (Table SX). The predominant macroinvertebrate orders were Decapoda (16.7%), Amphipoda (15.3%), Odonata (11.2%), and Isopoda (7.2%). The top three families represented 32.1% of the total community, including Decapoda: Palaemonidae (14.6%), Amphipoda: Hyalellidae (10.3%), and Isopoda: Aselidae (7.1%). There were no significant differences in Shannon index (χ^2^ = 8, *p* = 0.433) or Simpson (χ^2^ = 8, *p* = 0.433) diversity of macroinvertebrate communities collected among the sites.

## *Mycobacterium ulcerans* and *M. liflandii* VNTR genotypes were identified in the Southeastern US

**Figure 4.**
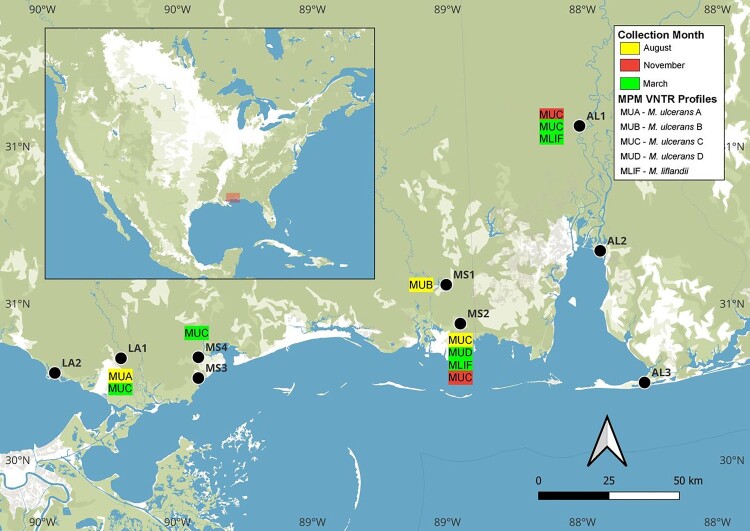
Distribution of *Mycobacterium ulcerans* and *M. liflandii* VNTR profiles in Southeastern United States (Mississippi, Louisiana and Alabama). Sites in Louisiana are shown as purple diamonds, Mississippi as orange diamonds and Alabama as black diamonds. Genotypes identified from August 2020 samples are highlighted in yellow, November 2020 highlighted in red, and March highlighted in green. MUA: *M. ulcerans* VNTR profile A; MUB: *M. ulcerans* profile B; MUC: *M. ulcerans* profile C, MUD: *M. ulcerans* profile D; Mlif: *M. liflandii* profile.

MU and *M. liflandii* VNTR genotype profiles were identified among 12 samples across sites and sampling times (Figure 4). Overall, MU genotypes A (1,1,1,2), B (3,1,1,2), C (3,1,2,2), D (1,1,2,2), and *M. liflandii* (1,2,2,1) were identified [3, 25]. Complete VNTR genotype profiles were identified in August 2020 from one filter sample from each of the MS1, MS2, and L1 sites, and two filters from AL1, matching MUB, MUC, MUA, and MUC and *M. liflandii*, respectively. One filter sample taken in November 2020 from MS2 and one from AL2 had complete profiles matching MUC. In March 2021, two filter samples from MS1 had complete profiles matching MUD and *M. liflandii*, one from MS4 matched MUC, and two from LS1 matched MUC. Remaining positive samples were negative, had incomplete profiles, or those that did not match known control profiles from MPM isolates.

## Discussion

Our study identified an expanded MPM range in Southern US samples across three sampled states, and multiple environmental matrices. Using specific molecular targets, we report MU/MPM persistence over time by measuring their spatio-temporal presence at sites previously sampled by Hennigan et al. in 2013 [14]. However, our work spans beyond Hennigan et al. (2013) [14] with more specific molecular targets, and VNTR matched profiles, and so provides important and novel evidence of MU and other MPMs in the US. The observed persistence suggests that these ecosystems continue to support MU and other MPMs over extended periods and harbour these mycobacteria long term rather than sporadic contamination events. The finding of a broader MPM distribution is consistent with MPMs as environmental sapronotic pathogens, and the presence of MU and other MPMs in the Southeastern US. Our results support that southern areas of the US harbour biomes that represent ecological conditions of the Neotropics and tropics known to support MU and other MPMs [5,9,14,16,27]. This biogeography helps to explain distributional patterns of these NTMs, as they are not confined by political borders and are likely found in other tropical and sub-tropical regions of the world.

Peak MPM detection was in August 2020, and was across all sites, followed by a significant decline in positivity and concentration during the subsequent sampling periods in November 2020 and March 2021, suggesting warmer summer months offer a more favourable environment for MPM persistence and proliferation, though rainfall patterns may have also driven effects, though not measured here. Three sites in November could not be sampled and missing November data for those sites could mean we potentially underestimated overall fall positivity at those sites (had they been sampled; they might have contributed additional positives). However, those sites (MS1, MS4, AL3) had moderate or low positivity in the other months, hence their absence in November likely did not change the overall finding of positivity decline from summer to fall. The fact that detection diminished in ensuing months could be attributed to changing environmental conditions, likely affecting MPM distribution [14,27-28]. These trends are consistent with the findings of Hennigan et al. (2013) who reported seasonal variation in IS2404 prevalence, with higher positivity during the summer months and a decline toward fall [14]. While our data suggest a seasonality component, additional longitudinal studies are necessary to confirm, as we only sampled during one seasonal year. Environmental conditions (i.e. temperature, pH, dissolved oxygen, and water flow) may play key roles in MPM population dynamics, as suggested by similar studies conducted in other regions [14,27-28]. For instance, Garchitorena et al. [29] in Cameroon demonstrated water temperature was a significant factor driving MU population dynamics. A similar influence may be important in the Southeastern US, where warmer temperatures during August may create an ideal habitat for MPMs. More broadly, this finding is consistent with another study by Kirschner et al. [30] who isolated and identified *M. avium*, *M. intracellulare*, and *M. scrofulaceum* (MIAS), other NTMs, from water, soil, aerosols, and droplets in the Southeastern US coastal plains, and reported higher MIAS numbers correlating with warmer temperatures. Warmer summer conditions likely create more favourable environments such as higher microbial growth rates favourable for MU. In our study area, summer water temperatures often exceeded 30 °C (26°C – 35°C) (Table WQ), whereas spring and winter waters were much cooler (mostly below 20 °C). Pearson’s correlation showed that water temperature appeared to strongly influence MPM positivity (r =  **+** 0.61) as higher temperatures were associated with a greater likelihood of detecting MPM, whereas cooler conditions corresponded to lower or absent MPM detection. Overall, these findings imply that warmer water temperatures tend to promote MPM positivity. MU grows best between 29°C and 33°C under laboratory conditions [1]⁣, so higher summer temperatures can accelerate its growth and increase its abundance in the environment. Additionally, summer waters had pH values closer to neutral, whereas some spring/winter waters were slightly more acidic. MU has a broad pH range, so the summer pH may further support the bacterium’s persistence [29]. Water pH did not show any clear relationship with MPM positivity in this study (r =  + 0.09) as MPM was found in both low-pH and neutral pH waters, indicating that pH in the observed range (approx. 5.0–8.0) neither strongly promotes nor prevents MPM occurrence. Specific conductance showed a slight negative association with MPM presence (r = −0.28), suggesting that more extreme conductivity such as very high salinity or mineral content might inhibit MPM.

Overall, our study findings align with previous studies identifying aquatic environments as MU and other MPM hotspots, suggesting these environments likely play a role in transmission dynamics of the organism [29]. However, the water column itself may be only a means of MPM dispersal as opposed to being the foremost MPM source, given that the majority of MPM concentrations from positive samples were low suggesting the detection of free DNA, rather than an association with a specific host reservoir.

It was also surprising that only two macrophyte biofilm samples were positive since macrophyte biofilm have been frequently positive in other studies in West Africa [30], South America [31] and Australia [32]. Furthermore, we found no macroinvertebrate taxa positive, contrary to what has been measured in other studies [33]. This was another surprising result and may reflect different food webs. For instance, in several MU studies in Africa, the macroinvertebrate communities were dominated by insects, with fewer crustaceans reported in collections from slow-moving waterbodies [25,34]. In the macroinvertebrate collections from the sites in the US many were dominated by shrimp compared to Africa waterbodies dominated by biting water bugs and beetles [34]. Shrimp are often considered collector-gatherers and scavengers of organic material, while biting water bugs and beetles are dominated by predators; such differences may reflect differences in trophic dynamics important for MU and other MPM in waterbodies of the US. Another difference in the present study compared to others from Africa [25,33-34] and South America [35] is that we did an external surface decontamination to remove nucleic acids and cells from the surface of the macroinvertebrates, which has not been performed aforementioned studies. One explanation may be that previous reports of MU/MPM associated with aquatic macroinvertebrates is from external colonization of the bacteria, and not internal associations, supporting hypotheses related to the importance of chitin to MU/MPM ecology [36]. However, targeted studies to test for external versus internal colonization are needed. Nevertheless, it is worthy to note that, in West Africa, similar sampling and genotyping protocols led to estimate MU concentration in water about two-fold higher than in present study [27]. This could thus suggest that MU concentration could remain too low in Southeastern US for being able to detect this bacterium in secondary-colonized habitats such as macrophyte biofilms and macroinvertebrates. Additional sampling in areas free from human infection cases are required for settling this point.

In fact, highest MPM concentrations came from two soil samples collected from MS1 in August 2020. Soil samples have showed low per cent positivity compared to other assayed matrices in other MU/MPM studies but with high concentrations [30]. Alkaline soil was also an environmental factor associated with increased odds of MU presence in Victoria, Australia [37]. A laboratory study showed MU remained viable in watery soil with a 4-log decrease in concentration over a four-month period [38]. More broadly, NTMs are widely distributed across a range of soil types which act as a reservoir for potential human and animal infection [28,30,39]. Presence and composition of NTM species in soil is dependent upon soil type, elevation, annual season and soil temperature, soil water content, vegetation, and habitat type [28,40]. Differences in these environmental parameters may drive MPM presence and abundance in soil in our study sites and are the subject of further examination by us.

Among positive samples, VNTR profiling identified *M. liflandii*, and several genotypes of MU, with MU genotype C being the most prevalent across all sampling sites and times. The dominance of genotype C may suggest this particular strain has adapted well to environmental conditions in the Southeastern US, similar to findings from BU endemic regions in West and Central Africa [4]. However, low MPM concentrations over time likely inhibited us from detecting complete profiles from more samples, although we did have some samples that showed incomplete profiles or those not matching any known profiles from MPM isolates.

**Figure 5.**
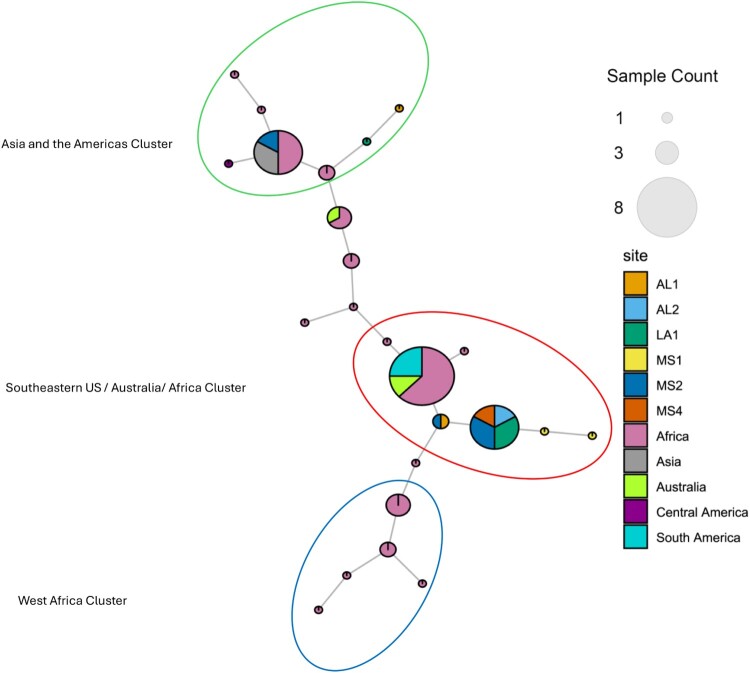
Minimum Spanning Tree of VNTR and VNTR-mycobacterial interspersed repetitive units (MIRU) profiles at four loci (Miru 1, Locus 6, ST1 and Locus 19). Samples include environmental samples collected from Southeastern United States (Mississippi (MS), Louisiana (LA) and Alabama (AL)), freezer stock controls from Africa (Ghana, Cote D’Ivoire, Cameroon and Benin), Asia (China and Japan), Australia and the Americas (Mexico, Surinam and French Guiana). Profiles from two previous studies were also included. Nodes represent similar genotypes. Number of individual samples are represented by the size of the node. Sites and Geographical origins of isolates are represented in the section of the node by colour. Three clusters were derived from this tree.

MST analysis revealed three distinct continental genotype clusters of MPMs (Figure 5), with a bulk of samples from Southeastern US (specifically from Mississippi and Alabama) clustering closely with isolates from Africa and Australia (Classical strains) [41] suggesting recent common ancestry and potential environmental persistence within aquatic reservoirs despite the absence of human BU disease in the US. Notably, these classical strains, known to cause significant disease burden in other regions, suggest a potential risk for human infection in Southeastern US if ecological conditions or human interactions with aquatic environments change. Another cluster also revealed some Southern US samples across all 3 states clustering closely with isolates from Asia and Mexico (Ancestral strains) [41]. This is not surprising as the Southeastern US is in close proximity to Mexico. The last cluster included isolates from West Africa.

Diversity and persistence of MPM genotypes in multiple locations points to the ecological adaptability of these organisms, and further highlights the potential risk for environmental transmission of mycobacteria in non-endemic regions. Although no human cases of BU have been reported in the US, other MPM infections with *M. pseudoshottsii* have been reported in the US, infecting fishes [42]. Though many of these MPM mycolactone congeners have not been directly associated with human BU disease, we previously discovered that MPM associated with fish disease (*M. pseudoshottsii*) may be pathogenic for humans; other groups have also reported that some non-human associated MPM mycolactone congeners were cytotoxic to human cells [6]. These MPMs also grow at temperatures lower than MU isolates that cause human infection under laboratory conditions, with differing doubling times, that complicates potential detection. Notwithstanding, environmental monitoring and continued surveillance are critical. Given the lack of knowledge related to MPMs in the US, we recommend additional research into developing environmental surveillance for both the pathogen and potential BU cases that may be going unreported. It is documented that BU cases are often un – or underreported in many parts of Africa [43], and likely elsewhere. In a systematic review of the global BU distribution, the authors concluded that BU distribution was uncertain and potentially wider throughout the world [43].

Finally, our data clearly support that MU and other MPM distribution is broader than reported disease and that some strains may be more likely to infect humans and animals than others as reported [16,22,25,27,33,44]. Other factors such as strain diversity, and human demography and behaviour are likely important in the epidemiology of MPM infection, though we cannot also rule out a recent expansion of MPM into these geographical areas. However, it is worthy of note that the few New-World isolates that have been included in MU/MPM phylogenetic studies are ancestral to the intensively used isolates collected in BU endemic areas (Africa and Australia), suggesting thus that the acquisition of virulence has taken place in the New World [45-47]. Meanwhile, addressing the variations in concentrations across environmental matrices in places where no human infection case had been reported allow an interesting comparison with previous data acquired in BU endemic areas. This allowed confirming across places with high and low MU/MPM environmental concentrations: (1) The occurrence of seasonal variations of MU/MPM concentration in soils than in freshwaters, and (2) Lower concentrations in freshwaters which tend to be more usually positive than soils, as if they represented dispersion factors of these bacteria. This also suggested that the macroinvertebrates only represent a secondary suitable habitat relative to soils and freshwaters, turning thus positive only in places where MU/MPM environmental concentrations are high.

Our data thus have widespread implications for the MU/MPM fields and for NTMs as a whole. Because MPMs and other NTMs can be maintained in environmental reservoirs [48], these mycobacteria frequently overlap with those of humans and domesticated animals [40]. Origin, spreading, and infection sources are also difficult to identify and are an epidemiological concern. Close contact with these sources through agriculture and recreational activities are considered risk factors, requiring transmission research to extend beyond person-to-person contact [49]. NTM elimination from environmental sources whenever possible and blocking related transmission are thus critical prevention issues, especially given that outdoor recreation participation continues to significantly rise. Importantly, warmer temperatures also provide a more favorable environment for proliferation and climate change may continue to increase and expand NTM infection risks globally [50-52]. Thus, continued studies defining the MPM environment and range in the broader context of NTM diversity will provide important data towards predicting and preventing exposure and infection. Our data reiterate the importance of continuous surveillance across varied environmental matrices implementing targeted interventions, especially in identified high-risk areas, and areas that are resource and infrastructure poor. Further longitudinal studies are necessary to evaluate seasonal patterns, environmental drivers, and the potential for human and animal infection in these regions.

## Supplementary Material

DOGBE_Supplementary Materials.docx

Supplemental VNTR mst.pdf

Table WQ.xlsx

Table SX.xlsx

Table CT.xlsx
