# Supplementary material for: Spatiotemporal distribution of Mycobacterium ulcerans and other mycolactone producing mycobacteria in southeastern United States
Source: Emerg Microbes Infect. 2025 Jun 17;14(1):2521853. doi: 10.1080/22221751.2025.2521853 (PMC12231298; doi:10.1080/22221751.2025.2521853)
Supplement: DOGBE_Supplementary Materials.docx [file TEMI_A_2521853_SM5196.docx]

**SUPPLEMENTARY MATERIAL**

**METHODS**


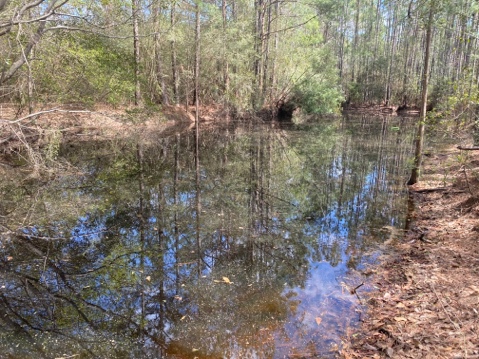

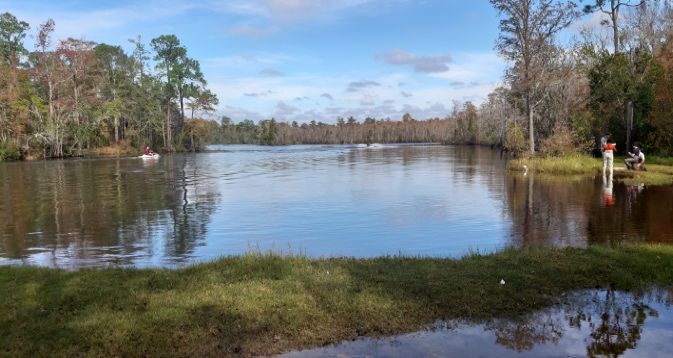

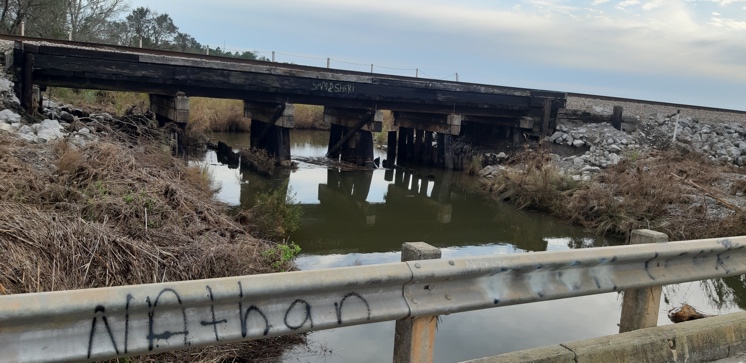

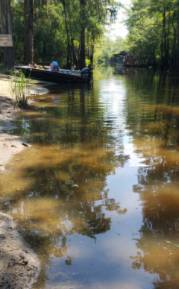


MS3 - Railroad Ave., Bay Saint Louis

MS1 - Hardy Waltman Road

MS2 - Pollock Ferry Rd Boat Ramp


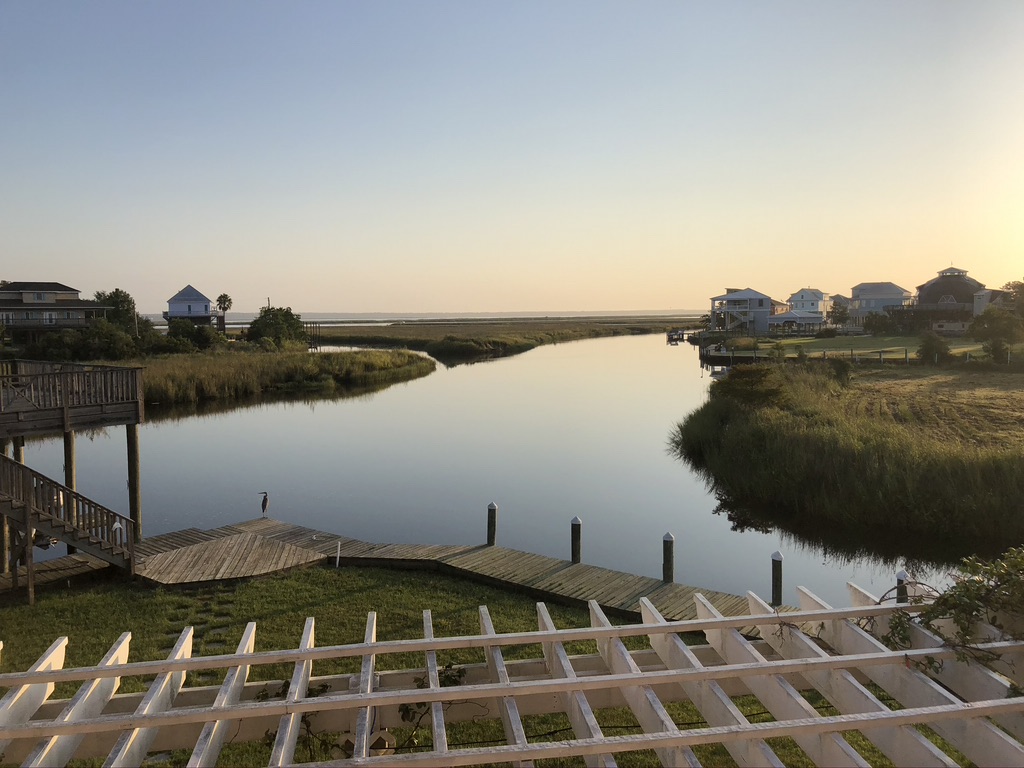

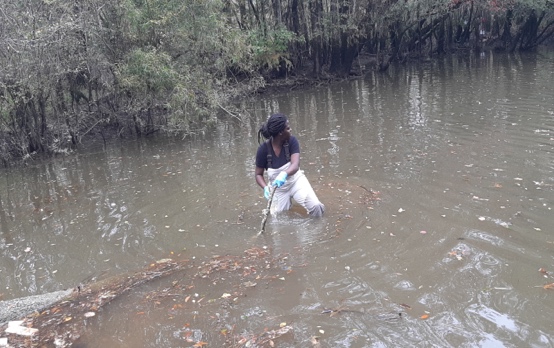

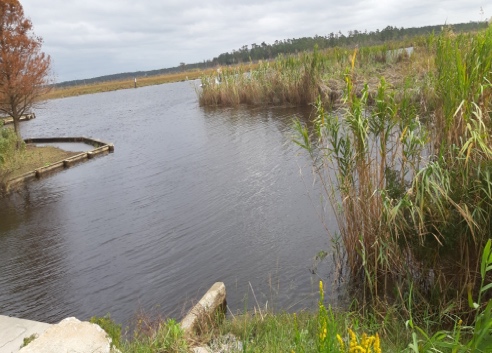


LA2 - Big Branch Marsh

LA1 - Davis Landing Road

MS4 - Tarpon Drive Boat Ramp


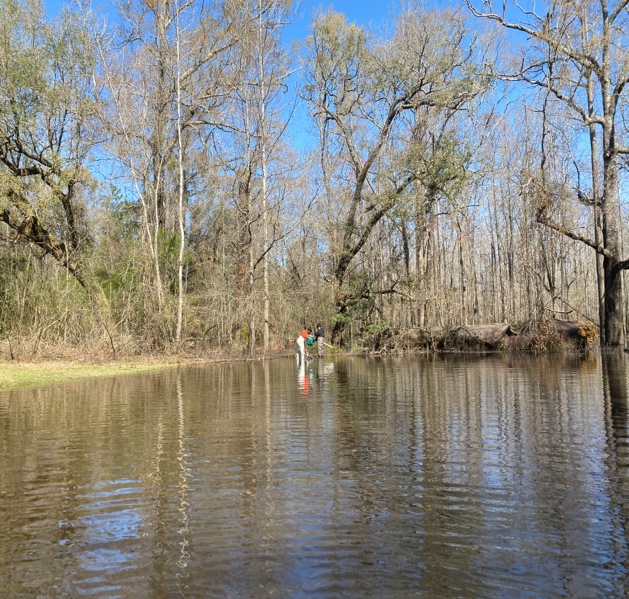

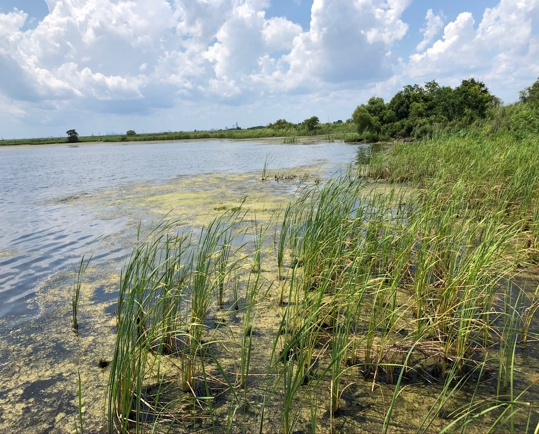

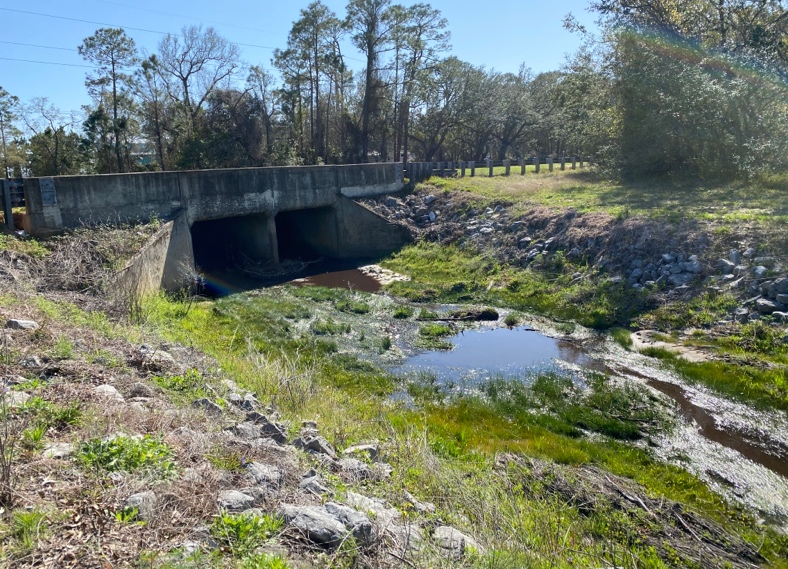


AL3 - Pinnacle Course Creek

AL2 - Meaher State Park

AL1 - Cedar Creek Landing

Figure S1: Representative images of sites for sampling water, macrophyte biofilms and soil. Samples were collected August, and November 2020 and March 2021. MS: Mississippi; LA: Louisiana; AL: Alabama.

**DNA Isolation, and PCR targeting Mycolactone producing mycobacteria**

DNA extraction was performed using reagents and methods previously described by Williamson, et al., (1)and detailed as supplementary material. Briefly, 750µL of lysis solution and 0.5mg of a 1:1 mixture of 0.5mm and 0.1mm glass beads (Research Products International) were added to samples (excluding invertebrates) in a 2mL screwcap tube (Millipore Sigma). Samples underwent homogenization in a beadbeater at 3,500 oscillations per minute for 45 seconds, 2-minute incubation on ice, and another 45-second homogenization cycle at 3,500 oscillations per minute. Samples were incubated at 65˚C for 20 minutes and then centrifuged for 2 minutes at 4˚C at 10,000xG. Resulting supernatant (400µL) was transferred to individual 1.5mL tubes containing 150µL potassium acetate and incubated at -20˚C for 1 hour. Samples were then centrifuged at 4˚C for 30 minutes at 10,000xG. Next, 1.2mL of guanidine hydrochloride was added to individual 2mL tubes, and each sample's supernatant was combined with the guanidine hydrochloride. Following thorough pipette-mixing, 700µL from each sample was transferred to individual spin filters (Epoch LifeSci Econospin) and centrifuged for 2 minutes at 10,000xG, with the resulting flow-through discarded. This process was repeated until all the sample passed through the spin filter. Subsequently, 500µL of wash solution was added to the filter, and samples were centrifuged for 2 minutes at 10,000xG, with the resulting flow-through discarded. The sample filters were further washed with 500µL of 100% ethanol and centrifuged for 2 minutes at 10,000xG. After centrifugation for 5 minutes at 10,000xG to remove residual ethanol, the spin filters were carefully transferred to new 2mL tubes. Elution solution (60µL) was added, and samples were incubated at room temperature for 10 minutes. Following the incubation, samples were centrifuged for 2 minutes at 10,000xG at room temperature. DNA concentrations were assessed using a Qubit 2.0, and protocol for High Sensitivity DNA detection.

DNA from invertebrates was isolated at Michigan State University using the QIAGEN DNeasy Blood & Tissue kit with a modified protocol. During each extraction, 15 mg/ml of lysozyme was added during the lysis step, and 100 mg/ml of RNase A was added immediately after the incubation. Prior to extraction, all insects were surface decontaminated using two 10% bleach washes followed by three sterile water washes and subsequently homogenized in TE buffer using a sterile microtube pestle.

**Quantitative PCR for Mycolactone Detection**

DNA samples were subjected to quantitative PCR using a Taqman probe with VIC fluorophore and primers targeting the ER as previously described (Table 1) (2). Standards were made by cloning the amplified ER gene from MU 1615 into a TOPO TA cloning vector and transforming DH5α *E. coli* cells prior to plasmid isolation. Plasmids were purified using a GeneJET Plasmid miniprep kit and quantified using Qubit 2.0. Serial dilutions of purified plasmid were used to generate the calibration curve for quantifying DNA samples. PCR runs were performed in 96-well plates in duplicates for samples and triplicates for standards with each well containing a final volume of 25 μL (12.5 μL Taqman environmental mastermix 2.0, 2.5 μL Taqman VIC probe, 1 μL forward primer, 1 μL reverse primer, 5 μL water and 3 μL template DNA). Appropriate positive and negative controls were included. Quantitative PCR was run on the C1000 touch thermal Cycler (BioRad CFX96) with conditions as follows: 2 min at 95 ^o^C then 10 min at 95 ^o^C followed by 40 cycles of 15 s at 95 ^o^C and, 1 min at 55 ^o^C. A sample was scored positive for MPM DNA if duplicate samples showed a cycle threshold of less than 38.0, with standard curve R^2^ $\geq$ 0.99 and a log linear slope within the ranges of – 2.9 and -3.6. Runs were repeated for duplicates with inconsistent results or if the above stated criteria were not met. A subset of samples representing all matrices and sites were also diluted or spiked to rule out inhibition. Mycolactone producing mycobacteria copies in genome units (GU) per sample were extrapolated and expressed as GU/sample.

**Variable Number Tandem Repeat (VNTR) Profiling and Intergenic Region Genotyping**

Samples where MPMs were detected were further subjected to VNTR profiling targeting four loci: MIRU1, locus 6, ST1, and locus 19. VNTR primer sequences (Table 1), PCR thermocycler conditions, controls, and downstream sequencing confirmation were performed as described by Williamson *et al* ^1^. Strain and regional specific controls were used for comparison. Banding patterns from all strains were compared against known controls.

**Table 1: Primer pairs used for the study**

| **Gene Target** | **Forward primer (5’-3’)** | **Reverse primer (5’-3’)** |
| --- | --- | --- |
| Locus 6 | GACCGTCATGTCGTTCGA | GACATCGAAGAGGTGTGG |
| MIRU 1 | GCTGGTTCATGCGTGGAA | GCCCTCGGGAATGTGGT |
| ST1 | CTGAGGGGATTTCACGACCAG | CGCCACCCGCGGACACAGTCG |
| Locus 19 | CCGACGGATGAATCTGTAGGT | TGGCGACGATCGAGTCTC |
| ER | ﻿ CGCCTACATCGCTTTGG | ATTGAATCGCAGCCATACC |
| ER-VIC Probe | ﻿VICCTGATCCATGCCGGC MGBNFQ | |

**REFERENCES**

1. Williamson H, Phillips R, Sarfo S, et al. Genetic diversity of PCR-Positive, culture-negative and culture-positive Mycobacterium ulcerans isolated from Buruli ulcer patients in Ghana. PLoS One. 2014;9(2):1–5.

2. Williamson HR, Benbow ME, Campbell LP, et al. Detection of mycobacterium ulcerans in the environment predicts prevalence of Buruli ulcer in Benin. PLoS Negl Trop Dis. 2012;6(1).
