# Supplementary material for: Spatiotemporal distribution of Mycobacterium ulcerans and other mycolactone producing mycobacteria in southeastern United States
Source: Emerg Microbes Infect. 2025 Jun 17;14(1):2521853. doi: 10.1080/22221751.2025.2521853 (PMC12231298; doi:10.1080/22221751.2025.2521853)
Supplement: Supplemental VNTR mst.pdf [file TEMI_A_2521853_SM5195.pdf]

```r
#install.packages(c("tidygraph", "ggraph", "ggplot2", "dplyr", "tidyr"))

library(ggraph)
library(tidygraph)
library(ggplot2)
library(dplyr)
library(tidyr)
library(RColorBrewer)
library(igraph)
library(tidygraph)
library(viridis)
library(scales)


#set working directory
setwd("~")

#import vntr data
vntr_data <- read.csv("VNTR_repeat_counts.csv", header = TRUE)
head(vntr_data)

rownames(vntr_data) <- vntr_data$LAB.CODE
vntr_data$LAB.CODE <- NULL

#collapse identical genotypes

vntr_matrix <- vntr_data %>% select(1:4)
vntr_data <- vntr_data %>%
  mutate(genotype_id = apply(select(., 1:4), 1, paste, collapse = "_"))

# Group and summarize
genotype_summary <- vntr_data %>%
  group_by(genotype_id) %>%
  summarize(count = n(), .groups = "drop")

# Extract unique genotypes
unique_genotypes <- vntr_data %>%
  distinct(genotype_id, .keep_all = TRUE) %>%
# select(-LAB.CODE) %>% # or whatever non-genotype columns you had and
 want to remove
 select(-STATE) %>%
 select(-Matrix) %>%
 select(-GENOTYPE) %>%
 select(-Month) %>%
 select(-Source) %>%
 select(-Site)%>%
 select(-Site2)
```

```r
# Remove the genotype_id column for distance calculations
geno_matrix <- unique_genotypes %>% select(-genotype_id)
rownames(geno_matrix) <- unique_genotypes$genotype_id

#create distance matrix from VNTR repeats
dist_mat <- dist(geno_matrix, method = "manhattan")

g2 <- graph_from_adjacency_matrix(as.matrix(dist_mat), weighted = TRUE,
 mode = "undirected")
mst_g2 <- mst(g2)

# Map sample counts to vertices
V(mst_g2)$count <- genotype_summary$count[match(V(mst_g2)$name,
 genotype_summary$genotype_id)]

# Use this for vertex size
V(mst_g2)$size <- sqrt(V(mst_g2)$count) * 5

#stitch to ggraph to use pie charts on nodes
#put sites and sample ID in a data frame
sample_site_data <- data.frame(
  sample_id = rownames(vntr_data),
  site = vntr_data$Site
)

#match sample IDs to genotypes
sample_to_genotype <- data.frame(
    sample_id = rownames(vntr_data),
  genotype_id = vntr_data$genotype_id
)

# Merge site data
site_counts <- sample_to_genotype %>%
  left_join(sample_site_data, by = "sample_id") %>%
  count(genotype_id, site)

# Normalize to proportions per genotype
site_props <- site_counts %>%
  group_by(genotype_id) %>%
  mutate(prop = n / sum(n))

# Convert MST to tidygraph
tg_mst <- as_tbl_graph(mst_g2)

# List of proportions per node
site_list <- site_props %>%
  group_by(genotype_id) %>%
  summarise(site_data = list(tibble(site = site, prop = prop)))
```

```r
# Add to graph
tg_mst <- tg_mst %>%
  left_join(site_list, by = c("name" = "genotype_id"))

#count totals per genotype
genotype_counts <- sample_to_genotype %>%
  count(genotype_id, name = "sample_count")

# Set up site colors
#for continents
desired_order <- c("AL1", "AL2", "LA1", "MS1", "MS2", "MS4",
                "Africa", "Asia", "Australia",
                 "Central America", "South America")

#for countries
#desired_order <- c("AL1", "AL2", "LA1", "MS1", "MS2", "MS4",
 "Australia", "Benin", "Cameroon", "China", "French Guiana", "Ghana",
 "Ghana and Cote D'Ivore", "Japan", "Mexico", "Surinam")

#order the sites for color assignement-I want our sites first
site_levels <- intersect(desired_order, unique(site_props$site))

#site_colors <- setNames(viridis(length(site_levels)), site_levels)

#site_colors <- setNames(viridis_pal(option = "D")(length(site_levels)),
 site_levels)

#library(colorspace)
#site_colors <- setNames(qualitative_hcl(length(site_levels), palette =
 "Dark 2"), site_levels)

#for continents
# Okabe-Ito palette (colorblind-friendly)
okabe_ito <- c(
  "#E69F00", "#56B4E9", "#009E73", "#F0E442",
  "#0072B2", "#D55E00", "#CC79A7", "#999999",
  "#ADFF2F", "#8B008B", "#00CED1"
)

#for countries
#okabe_ito <- c(
 # "#E69F00", "#56B4E9", "#009E73", "#F0E442",
 # "#0072B2", "#D55E00", "#CC79A7", "#000000",
#  "#999933", "#66CCEE", "#228833", "#CCBB44",
 # "#4477AA", "#AA3377", "#EE6677", "#BBBBBB"
#)
```

```
#maintain site order with colors
site_colors <- setNames(okabe_ito[1:length(site_levels)], site_levels)

# Extract layout positions for each node (genotype)
layout <- create_layout(tg_mst, layout = "kk")

# Merge counts with layout and pie angles
pie_data <- layout %>%
  left_join(genotype_counts, by = c("name" = "genotype_id")) %>%
  left_join(
    site_props %>%
      group_by(genotype_id) %>%
      arrange(site) %>%
      mutate(
        end = 2 * pi * cumsum(prop),
        start = lag(end, default = 0)
      ),
    by = c("name" = "genotype_id")
  ) %>%
  mutate(radius = scales::rescale(sample_count, to = c(0.03, 0.25))  #
 scale radius

pie_data$site <- factor(pie_data$site, levels = desired_order)

#now plot the graph
ggraph(layout) +
  geom_edge_link(color = "grey70") +

  # Point layer for size legend
  geom_node_point(
    data = pie_data %>% distinct(name, x, y, sample_count, radius),
    aes(x = x, y = y, size = radius^2),
    color = "grey30",
    alpha = 0.5  # invisible, just for legend
  ) +

  # Pie nodes
  geom_node_arc_bar(
    data = pie_data,
    aes(
      x0 = x, y0 = y,
      r0 = 0,
      r = radius,
      start = start,
      end = end,
      fill = site
    )
  ) +
```

```
  scale_size_continuous(
    name = "Sample count",
    breaks = c(0.005625, 0.03125, 0.0625),  # these are radius^2 values
DOESN'T WORK FIX IN PHOTOSHOP
    labels = c("1", "3", "8"),           # actual sample counts
    guide = guide_legend(override.aes = list(
      shape = 21, fill = "grey80", color = "grey30", stroke = 0.5
    ))
  )+

  scale_fill_manual(values = site_colors) +
  theme_void() +
  theme(legend.position = "right")
```
